# Neoantigen-Specific Adoptive Cell Therapies for Cancer: Making T-Cell Products More Personal

**DOI:** 10.3389/fimmu.2020.01215

**Published:** 2020-06-26

**Authors:** Valentina Bianchi, Alexandre Harari, George Coukos

**Affiliations:** ^1^Department of Oncology, Lausanne University Hospital, Ludwig Institute for Cancer Research, University of Lausanne, Lausanne, Switzerland; ^2^Center of Experimental Therapeutics, Department of Oncology, University Hospital of Lausanne (CHUV), Lausanne, Switzerland

**Keywords:** cancer immunotherapy, adoptive cell therapy (ACT), tumor-infiltrating lymphocyte (TIL), neoantigens, enrichment

## Abstract

Mutation-derived neoantigens are taking central stage as a determinant in eliciting effective antitumor immune responses following adoptive T-cell therapies. These mutations are patient-specific, and their targeting calls for highly personalized pipelines. The promising clinical outcomes of tumor-infiltrating lymphocyte (TIL) therapy have spurred interest in generating T-cell infusion products that have been selectively enriched in neoantigen (or autologous tumor) reactivity. The implementation of an isolation step, prior to T-cell *in vitro* expansion and reinfusion, may provide a way to improve the overall response rates achieved to date by adoptive T-cell therapies in metastatic cancer patients. Here we provide an overview of the main technologies [i.e., peptide major histocompatibility complex (pMHC) multimers, cytokine capture, and activation markers] to enrich infiltrating or circulating T-cells in predefined neoantigen specificities (or tumor reactivity). The unique technical and regulatory challenges faced by such highly specialized and patient-specific manufacturing T-cell platforms are also discussed.

## Introduction

In the new age of personalized immune-oncology, tumor-infiltrating lymphocytes (TILs) generated from surgical resections, expanded *in vitro* and adoptively transferred, provide a unique opportunity to harness the specificity and diversity of the patient's endogenous T-cell repertoire. Building on the promising clinical outcomes achieved by TIL therapy in melanoma and cervical cancer (1, 2), efforts are now made to generate even more tailored T-cell products with predefined antigen specificities and, potentially, with enhanced *in vivo* tumor reactivity. The success of personalized adoptive cell therapies (ACTs) is therefore tightly linked to the identification of tumor-associated antigens, which are essential for tumor control.

Against this background, cancer neoantigens deriving from private mutations represent an ideal class of cancer antigens to target in that they are highly tumor-specific by nature, therefore reducing the potential induction of central and peripheral tolerance (3, 4). Most studies predominantly focus on single-nucleotide variants (SNVs) when referring to immunogenic tumor-specific mutant peptides; however, small insertions and deletions (indels), gene fusions, and posttranslational modifications (such as phosphorylation or glycosylation, which often alter the protein structure and function) have also been recognized as important neoantigen sources, therefore expanding the plethora of potential targets for cancer immunotherapy (5–9). Furthermore, non-canonical major histocompatibility complex (MHC) peptides derived from annotated noncoding regions are emerging as critical immune regulators across cancer types and able to elicit tumor-specific T-cell responses (10, 11).

Neoantigen discovery is a multistep process performed on a patient-specific basis by cutting-edge preclinical pipelines integrating variant calling, *in silico* filtering, and immunogenicity evaluation, leading to private (and shared) neoantigen candidates (12–14). Briefly, mutations are called by whole-exome or whole-genome sequencing of tumor vs. germline DNA, are further filtered by *in silico* prediction algorithms and potentially tumor RNA sequencing immunopeptidomics, primarily taking into account peptide-MHC binding affinity and RNA expression as well as direct identification (15). Additional peptide features, such as stability, clonality, cleavage scores, variant allele frequency, dissimilarity to self, or mutation coverage, are now also taken into account as potential determinants of immunogenicity (16–18). The downstream number of short-listed neoepitopes varies among patients and tumor types and is further greatly reduced following cellular immunogenicity evaluation. Depending on the chosen experimental strategy, prioritized neoepitope candidates are synthesized in the form of short or long peptides, or mRNA encoding mutations, and screened for T-cell reactivities from patients' blood or tumor samples. In this context, functional assays [such as interferon (IFN)-γ ELISpot and CD137 assay] as well as peptide MHC (pMHC)-multimer direct stainings are typically used as sensitive readouts. Of note, cellular interrogation requires a significant number of patients' samples and often includes, prior to screening, a round of antigen-specific T-cell expansion with candidate neoepitope pools, which may alter the original clonotypes' composition.

Despite the variable mutational load across different human malignancies (19) and the technical challenges, tumor-infiltrating, as well as circulating, neoantigen-specific CD8^+^ and CD4^+^ T-cells have now been identified and characterized in several tumor types (20–25). Early clinical data also suggest that neoantigen load has a predictive role in patient response to checkpoint blockade and TIL ACT immunotherapy (26–29).

Bulk infiltrating T-cell populations can be very heterogeneous, and the frequency of private (and shared) tumor-associated antigen specificities is generally low (20, 21, 30). Dissection of melanoma and colorectal and lung cancers has highlighted that a significant fraction of TILs can contain antiviral CD8^+^ T cells [such as Epstein-Barr virus (EBV)- and cytomegalovirus (CMV)-specific], extending observations that many tumor infiltrates may be in fact not tumor-specific (30–32). A study by Scheper et al. (33) has assessed the intrinsic tumor reactivity of TILs in melanoma and ovarian and colorectal cancer, demonstrating how indeed only a small fraction of the intratumoral CD8^+^ T-cell receptor (TCR) repertoire is able to recognize autologous cancer cells. Yet, the frequency of CD8^+^ in TILs correlates with favorable prognosis, and increasing evidence has shown how a relatively limited set of neoantigen-specific T-cells from melanoma TILs can mediate tumor recognition, despite the tumor cells harboring hundreds of somatic mutations (34–37). Collectively, these data suggest that enriching TIL infusion products for a few T-cell clonotypes specific for key immunogenic neoantigens could guide more effective antitumor responses *in vivo*.

One might argue that the need for available resected tumor specimens, from which infiltrating T-cells are isolated *ex vivo*, limits a broader application of standard TIL therapies to other tumor types. In this regard, Cohen et al. (22) first provided a simplified and noninvasive blood-based strategy as an alternative to current TIL production by demonstrating that neoantigen and self-antigen reactive T-cells can be reliably isolated from the peripheral blood of melanoma patients. Detection of neoantigen-specific CD8^+^ and CD4^+^ lymphocytes from peripheral blood has been subsequently described in patients with relatively low tumor mutation burden, such as ovarian and gastrointesinal cancers (20, 38–40). However, circulating neoantigen-specific T-cells share with their infiltrating counterpart very low detection frequencies (ranging from 0.5 to 0.002%) (20, 22, 37, 41, 42), hence the need for specific enrichment strategies. Of note, novel evidence has shown that the patient neoantigen-reactive CD8^+^ TCR repertoire can be largely discordant (in terms of specificity and functional avidity) between circulating and infiltrating T-cells in ovarian cancer patients (20). In particular, neoantigen-specific TILs exhibited on average higher functional avidity than their peripheral blood lymphocyte (PBL) counterpart. Further studies are therefore required to assess whether PBL and TIL cultures can be an equally suitable source for successful personalized T-cell therapies. Of note, it has also been shown that non-tolerized CD8^+^ T-cell repertoires of healthy donors were able to specifically recognize neoantigens which were ignored by tumor-infiltrating T cells in melanoma patients (43, 44).

**Figure 1:**
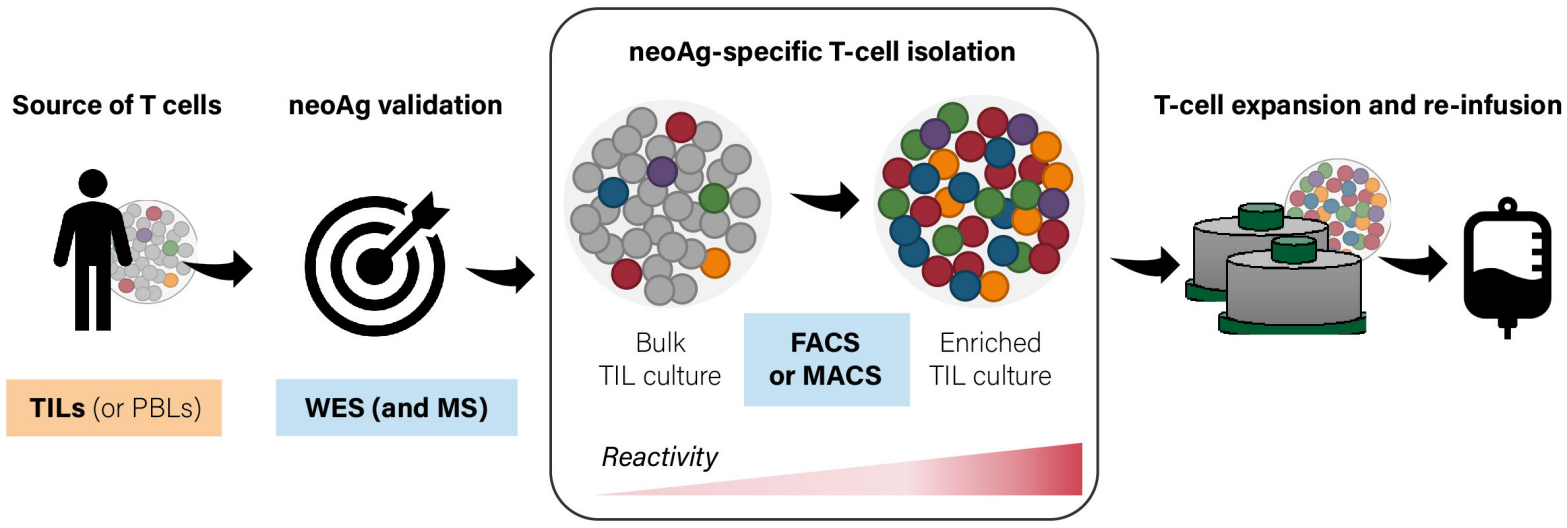
General workflow for personalized enrichment of antigen-specific T-cells from bulk tumor-infiltrating lymphocyte (TIL) [or peripheral blood lymphocyte (PBL)] cultures. T-cells can be isolated from the patient's infiltrating or circulating lymphocyte populations. Following neoantigen discovery and validation, antigen-specific T-cells are enriched by bulk cultures and expanded *in vitro* to meet the numbers required for reinfusion. FACS, fluorescence-activated cell sorting; MACS, magnetic bead-activated cell sorting; WES, whole-exome sequencing; MS, mass spectrometry.

**Figure 2:**
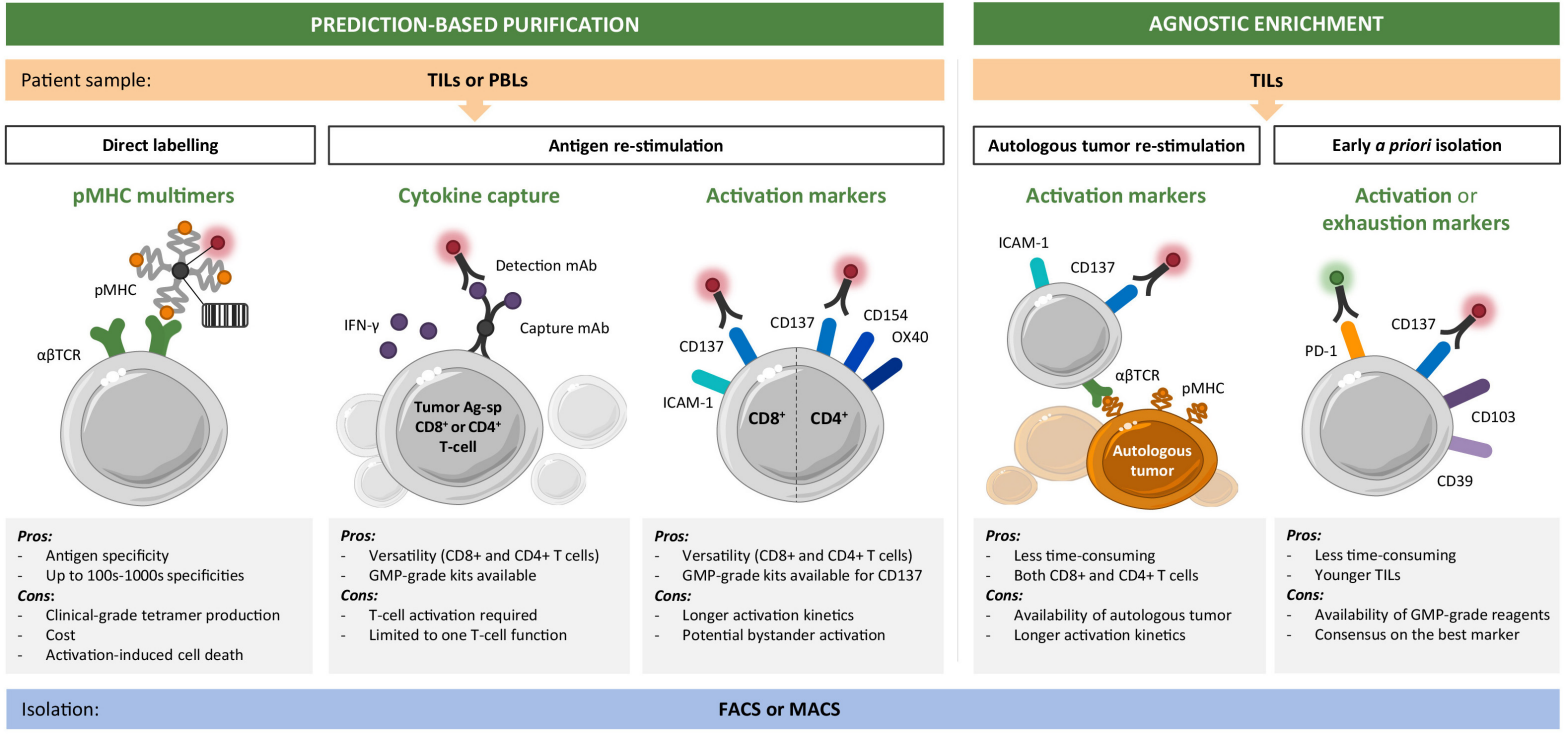
Toolset for personalized enrichment of T-cell infusion products. Current technologies can be grouped into neoantigen-specific purification strategies, which rely on predictions and predefined epitope selection, and agnostic enrichment strategies based on coculture with autologous tumor or *a priori* identification of tumor-reactive T-cells. The main advantages and disadvantages of each approach are listed. CD8^+^ (and CD4^+^) T-cells of interest can be isolated starting from tumor infiltrating lymphocyte (TIL) or peripheral blood lymphocyte (PBL) cultures by fluorescence-activated (FACS) or magnetic bead-activated (MACS) cell sorting.

Taken together, the selective enrichment of bulk TIL or PBL cultures for private (and shared) tumor-antigen specificities may improve the response rates achieved to date by adoptive T-cell therapies. One can envision highly personalized and specialized platforms, which integrate tumor-antigen identification and the generation of T-cell infusion products with a predefined reactivity composition (Figure 1). Here, we provide an overview of the current toolbox of technologies for the tailored enrichment of T-cell products in tumor-specific reactivities, addressing main advantages and disadvantages of individual approaches. A first general distinction can be made between techniques which require T-cells to be reactivated with cognate antigen (or autologous tumor) prior to downstream readout and separation (i.e., cytokine production or surface activation marker expression), and methods (such as pMHC multimer-based labeling) in which unstimulated antigen-specific T-cells can be directly selected. Different ways to restimulate antigen-specific T-cells are beyond the scope of this review and have been extensively discussed elsewhere (4, 45). A second distinction can be made between antigen-specific purification pipelines based on predefined specificities of interest and requiring a thorough antigen discovery phase and tumor reactivity-based pipelines which aim for a more “agnostic” enrichment in that they do not require *a priori* target prediction and identification (Figure 2). The two main technologies for cell isolation are fluorescence-activated and magnetic bead-activated cell sorting (FACS and MACS, respectively), both of which are extensively employed in preclinical research environments. Finally, we will address some of the challenges and limitations that such individualized T-cell manufacturing platforms necessarily entail for clinical application from both a technical and regulatory point of view.

## Current Toolset for the Enrichment of Predefined Neoantigen Specificities

### Peptide Major Histocompatibility Complex-Based Strategies

Labeling of a specific TCR by means of fluorochrome-conjugated pMHC multimers allows to directly identify CD8^+^ T-cell reactivities without restriction to functional parameters. MHC-based reagents have rapidly evolved from single fluorescent-labeled pMHC tetramers to increasingly advanced and optimized staining protocols with higher detection sensitivity (46, 47). For example, the screening of multiple T-cell reactivities can be achieved by combinatorial multimer staining either assigning a unique binary color code to each antigen specificity (48, 49) or using a high number of possible fluorochrome combinations (50). Several groups have speculated a possible clinical implementation of MHC multimer-based approaches in order to screen samples and generate neoantigen-enriched therapeutic cellular products (22, 51–53).

Alternative pMHC multimeric reagents, such as Streptamers and NTAmers, are built on reversible complexes and can therefore rapidly dissociate in the presence of biotin or imidazole, respectively (54–56). Antigen-specific T-cell staining with reversible multimers not only improves conventional pMHC reagents by reducing activation-induced T-cell death but also allows pMHC monomer dissociation kinetic measurements which have been shown to correlate with T-cell functionality (55, 57, 58). These technologies could therefore further aid in the precise selection of the “fittest” T-cell clonotypes within a single antigen specificity.

A more recent addition to the pMHC multimer portfolio is represented by a different labeling whereby DNA bar code tags are attached to the multimer scaffold. T-cells are collectively sorted based on one fluorescence, and distinct pMHCs are retrospectively revealed by means of large-scale sequencing of cognate bar codes (59). As a result, up to thousands of unique specificities can be potentially identified simultaneously, paving the way for high-throughput platforms and downstream applications such as TCR redirected T-cell therapy (60, 61). Of note, state-of-the-art microfluidic devices can help with potential sample size limitation by allowing *on-chip* detection and manipulation of multimer-sorted neoantigen-specific T cells for downstream analysis and applications (38).

If on one hand, pMHC-based strategies have facilitated the characterization of complex T-cell repertoires, on the other, they present some limitations for clinical application. First of all, the MHC restriction of the antigenic peptide has to be well-characterized; they do not provide information on T-cell function and are limited for CD4^+^ T-cell isolation (discussed below). Most importantly, given that pMHC multimer production is quite time-consuming and has to be manufactured under Good Manufacturing Practice (GMP) conditions, generation of a library of pMHC multimers on a patient-specific basis may be cost prohibitive for clinical implementation. In this regard, UV-based peptide exchange technologies (62) could aid the rapid engineering and manufacturing of multiple distinct pMHC reagents for individual patients.

### Cytokine Capture

Cytokines secreted by previously activated T-cells can be retained on the cell surface via a capture matrix, allowing the molecules detection and the isolation of viable antigen-specific T-cells via MACS (63, 64). In particular, IFN-γ secretion by activated CD8^+^ (and CD4^+^) T-cells has long been associated with effective tumor recognition and used as a functional readout to detect tumor-reactive T-cells (65, 66). However, there are only a couple of examples of preclinical isolation of tumor-specific T-cells by means of IFN-γ capture (65). Jedema et al. (67) describe a strategy to isolate leukemia-reactive CD8^+^ (and CD4^+^) T-cells upon specific IFN-γ secretion to be used for adoptive transfer. Another group has reported a GMP-grade isolation of protocol of polyclonal and polyfunctional antigen-specific T-cells from healthy donor PBLs, by IFN-γ labeling followed by FACS, using NY-ESO-1 as a model system (68). Taken together, fully automated IFN-γ-based T-cell enrichment procedures are commercially available and could be more easily implemented in a clinical pipeline. However, cytokine production is known to be restricted to certain T-cell subsets; therefore, the enrichment of antigen-specific T-cell frequencies uniquely based on cytokine secretion profile might be incomplete.

### Activation Markers

An alternative approach to direct labeling and cytokine detection is the use of activation-induced surface markers, which are upregulated upon antigen-specific TCR engagement. Expression of some of these markers is independent of cytokine production or T-cell phenotype, therefore potentially allowing the capture of the total pool of functional and reactive T-cells. Several surface markers have been suggested over time; however, only a limited number has been selected and extensively characterized because of reduced bystander activation, high specificity, and upregulation kinetics (69, 70).

The tumor necrosis factor receptor (TNFR) family member CD137 (or 4-1BB) has been initially characterized as a specific marker of TCR-induced activation of viral-specific CD8^+^ T-cells (70–72). Following antigen-specific stimulation, CD137 is upregulated on CD8^+^ T-cells, allowing the detection of viable antigen-specific T-cells. CD137 surface expression is now being extensively used as a marker to detect shared as well as neoantigen-reactive circulating and infiltrating T-cells, in combination with standard IFN-gamma ELISPOT screening (34, 42, 73–75). For example, Parkhurst et al. (73) isolated CD137^+^ TILs by FACS following restimulation with dendritic cells (DCs) transfected with mutation-encoding RNA and showed that the expanded CD137^+^ fraction had indeed been enriched in neoantigen-specific T-cells. At this point in time, anti-CD137 and anti-IFN-γ are among the few clinical-grade commercially available antibodies for the selection of antigen-specific T-cells; several companies though now provide custom monoclonal antibody development and conjugation under GMP guidelines.

On a different note, structural changes of activated integrins upon early TCR engagement can be exploited as the inside-out signal to detect functional T-cells (76). Dimitrov et al. (76) have successfully applied this strategy in order to monitor viral-specific T-cell responses within minutes, thereby stressing the advantageous very short incubation time compared to other activation markers. A parallel assessment of pMHC multimer and activation-based sorting would be highly informative in highlighting whether distinct markers are able to isolate overlapping populations of heterogeneous antigen-specific T-cells.

## CD4^+^ Neoantigen T-Cell Responses

Screening of naturally occurring or induced neoantigen T-cell responses in patients with solid tumors has provided evidence that both CD8^+^ and CD4^+^ T-cells recognize private mutated epitopes (24, 77–80). Furthermore, a number of single-patient case reports seem to indicate that neoantigen-specific CD4^+^ T-cells can mediate therapeutic immune responses to tumors (36, 81–83). A breakthrough paper by Tran et al. (36) provided the first demonstration of clinical activity of neoantigen-specific CD4^+^ T-cell infusion in a metastatic cancer patient.

Screening for MHC class-II-restricted T-cell has been long under-appreciated because of the limited accuracy of neoantigen prediction algorithms (84, 85). However, rapidly improving prediction tools for MHC class-II ligands (86–90) and the notion that TIL cultures can include a substantial fraction of functional CD4^+^ T-cells calls for flexible strategies to enable the enrichment of both CD4^+^ and CD8^+^ reactive compartments from bulk populations and downstream therapeutic infusion. In the framework of technologies validated for the CD8^+^ counterpart, MHC class-II multimers have so far progressed at a lower rate because of technical issues with recombinant pMHC class-II heterodimer production, and the assumption that CD4^+^ TCR binding affinity to cognate pMHC is significantly lower (91–93). On the other hand, activation marker upregulation following antigen restimulation offers the advantage of capturing cytokine-independent and heterogeneous CD4^+^ T-cell responses (71, 94). Indeed, CD137 may allow the capture of both CD4^+^ and CD8^+^ functional T-cells with high specificity (69, 95). However, a few publications have described the use of alternative TCR-dependent surface markers such as CD154 and CD134 (or OX-40) to detect neoantigen-specific CD4^+^ T-cells (34, 74, 75). A comprehensive comparison of activation-induced marker assays has yet not been investigated. In addition, care should be taken in discriminating regulatory T-cell from effector antigen-specific CD4^+^ T-cells when exploiting activation markers. For instance, the inverse expression of CD137 and CD154 has been described to discriminate between activated regulatory and effector CD4^+^ T-cells *ex vivo* (96).

### Agnostic Enrichment of Tumor-Reactive Tumor-Infiltrating Lymphocytes

The identification and validation of patient-specific immunogenic neoantigen specificities require advanced technologies (such as high-throughput sequencing, mass spectrometry, and synthetic peptide production) and adds complexity and time (several weeks to months) to an already labor-intensive TIL production pipeline. Less time-consuming and unbiased methods are therefore being evaluated to generate patient-specific T-cell products, which are clinically feasible for adoptive transfer.

### Coculture With Autologous Tumor

Using autologous tumor cells as targets circumvents the need for screening of immunogenic private or shared tumor antigens, while presenting to T-cells the complete range of naturally presented tumor antigens. In TIL production history, IFN-γ secretion has been exploited to prescreen which tumor fragments to expand: only TILs showing tumor reactivity above a predefined cutoff value were selected for downstream expansion and infused (97).

The role of activation marker CD137 was initially investigated by Ye et al. (98) for the quick and sensitive enrichment of tumor-reactive TILs from ovarian cancer and melanoma patients. The authors showed that CD137^+^-sorted TILs demonstrated increased reactivity against shared antigens following overnight incubation in the presence of MHC-matched tumor cell lines. Importantly, the CD137-enriched fraction resulted in enhanced *in vitro* and *in vivo* antitumor reactivity (98). Seliktar-Ofir et al. (99) presented proof of concept of a GMP-compatible CD137-based separation method for personalized adoptive cell therapy. Melanoma TILs were sorted by MACS based on CD137 surface upregulation following overnight coincubation with autologous tumor cultures (99). CD137^+^ TIL populations showed increased *in vitro* antitumor reactivity and contained a higher fraction of neoantigen and shared tumor antigen-specific T-cells when compared to the starting unseparated cultures. This approach might be limited by the establishment of autologous primary tumor cell lines, for which the success rate can be very low in tumors other than melanoma. In this regard, Dijkstra et al. (100) have presented a proof-of-concept study in which tumor-reactive T-cells from non-small-cell lung cancer and colon rectal cancer patients can be obtained by coculturing autologous PBLs with matched tumor epithelial organoids. Organoids are 3D cultures of primary solid tumors and can be established with a higher success rate from very limited amounts of tumor biopsies or surgical resections.

Considering the importance of costimulation in the context of successful tumor-specific T-cell activation, antigen presenting cells (APCs) should also be taken into account when establishing *ex vivo* cocultures of T cells and autologous tumor. The combination of natural or artificial APC with tumor lysate preparations can provide a wide array of tumor antigens in a more physiological costimulation context, therefore boosting the downstream antitumor activity of adoptively transferred T cells. Initial protocols used *ex vivo-*derived autologous DCs, stimulated with a defined maturation cocktail and pulsed with whole tumor lysate, to preferentially expand TILs to treat patients with melanoma (101–103). However, such strategies introduced additional time and numerous cytokines required for DC cell generation and maturation and prompted the quest for easily tailored and artificial APC (aAPC) platforms. Clinical-grade aAPCs have now limitless application potential: they can be coated with any number of costimulatory molecules (such as CD80, CD86, and CD137L) and membrane-bound cytokines to elicit rapid and improved TIL activation (104–106).

### *A Priori* Enrichment of Tumor-Reactive Tumor-Infiltrating Lymphocytes

Efforts from several groups are focusing on improving the *a priori* identification of tumor-reactive TILs solely based on phenotypic profiling. The rationale behind this strategy lies in the fact that naturally occurring tumor-reactive TILs are chronically exposed to their cognate antigen in the tumor site, therefore expressing a defined set of surface activation- and/or exhaustion-associated markers, providing the opportunity for their direct isolation.

Initial evidence suggested that preselection of melanoma-infiltrating or peripheral blood T-cells by PD-1 expression prior to expansion could directly enrich tumor-reactive T-cells (39, 107). In another study, CD137 was identified as a better marker than PD-1 for the prospective selection of naturally occurring tumor-reactive fresh TILs in ovarian cancer (98). Building upon these previous works, a defined set of tissue residency markers (such as CD103, or integrin αE), necessary for recruitment and retention of TILs in the tumor site, has been suggested as a prospective marker of TIL tumor reactivity (108–110). Duhen et al. (111) have shown that co-expression of CD103 and CD39 further enriches the TIL population for tumor-reactive CD8^+^ T-cells. CD103^+^ CD39^+^ TILs were sorted from tumor digests and expanded *in vitro*, resulting in increased cytotoxicity toward autologous tumor cells when compared to the respective single positive populations (111).

## Challenges and Limitations to Consider for Clinical Implementation

Compared to traditional biological molecules, personalized adoptive T-cell platforms are developed on a patient-specific basis, therefore presenting unique challenges not only for preclinical developers and manufacturers but also for regulatory authorities and healthcare providers. Starting from the initial step of private tumor antigen discovery and validation, throughout TIL (or PBL) enrichment and *in vitro* scale-up expansion, individual processes, facilities, and technologies must be carefully reviewed and adjusted according to clinical requirements. Indeed, specific regulations may differ slightly among countries and regions, but most challenges and limitations linked to clinical implementation are shared.

### Isolation Phase

Starting from the isolation step itself, one has to consider not only the technical aspects of the sorting strategy but more importantly its compatibility with regulatory requirements. The choice of a FACS or MACS-based enrichment depends on several factors, including the number of cells in the source material (TILs or PBLs), the relative frequency of antigen-specific cells within, the level of purity needed for the final product. On one hand, magnetic beads are of lower technical complexity and clinical-grade isolation kits are already commercially available, on the other, FACS separation performs multiparameter analysis of single cells, achieving resolution and purity levels, which are not always possible by MACS. In addition, FACS analysis can characterize in real time the sorted bulk T-cell population (in terms of identity and purity), as a first in-process quality control. However, FACS is still not routinely applicable under GMP conditions, which require single-use and a closed fluidic system for clinical implementation.

### Expansion Phase

As the absolute cell counts of neoantigen-specific T-cells after isolation are extremely low for direct reinfusion, a rapid expansion procedure (namely, REP) of sorted cells is typically performed with allogeneic irradiated feeder cells in the presence of high-dose interleukin (IL)-2 and anti-CD3 (97). Depending on the yield, the best scale-up closed-system expansion devices and culture conditions can be optimized to meet the numbers required for the adoptive transfer (typically in the order of 10^9^ cells per patient). Several distributors supply culture bags or gas-permeable flasks, sterile tubing accessories, and welding to facilitate the conversion of research protocols to GMP closed manufacturing processes, where the risk of cross contamination has to be minimized.

Absolute numbers aside, critical parameters for a successful ACT is ensuring that the final TIL product has maintained purity, TCR clonal diversity, and tumor reactivity following *in vitro* expansion. Indeed, during the REP phase, there can be interclonal competition resulting in an increased or decreased frequency of given specificities of interest compared to the starting culture. In this sense, enriching for tumor-specific T-cells at appreciable frequencies prior to up-scale would higher the chances of obtaining a final TIL product with adequate tumor reactivity upon infusion. The extensive expansion can also drive progressive T-cell differentiation and phenotype changes which may affect TIL *in vivo* persistence, homing, and proliferative capacity shortly after transfer, as these cells reencounter cognate antigens within the tumor microenvironment (112–115). Increased TIL proliferation and reactivity toward autologous tumor have been recently reported in a study introducing CTLA-4 blockade *in vitro* during the initial TIL pre-REP from ovarian tumor fragments (26). As mentioned previously, cytokines used during the *in vitro* manufacturing of the product can also significantly affect TIL immune profiles. Alternative cytokines to standard IL-2 have been tested during TIL REP (114) or during initial priming period (e.g., IL-21) (116, 117).

An additional aspect to consider is that TILs may fail to perform their expected therapeutic effector functions upon infusion due to activation-induced cell death (AICD) and exhaustion; both are peripheral tolerance mechanisms restricting an escalating, therefore potentially damaging, immune response. Melanoma TILs undergoing intense polyclonal TCR stimulation during REP have been shown to be more sensitive to AICD when cocultured *in vitro* with autologous tumor, whereas “younger” and less differentiated TILs are less susceptible and have a better *in vivo* tumor control (118–121). In a similar fashion, alternative costimulatory pathways, such as through CD137 via the addition of agonist antibodies during or following REP, can increase the polyclonal expansion of infiltrating or circulating CD8^+^ TILs while preserving their responsiveness (122–124).

Overall, how infused antigen-specific TIL clonotypes persist *in vivo* and respond to tumor antigen restimulation upon transfer and how their gene signature correlates to clinical benefit should be studied systematically on a larger number of patients receiving the same infusion regimen.

### General Improvements

Further pre-sensitization approaches could help increase neoantigen-specific T-cell frequency in starting TIL (or PBL) cultures and facilitate downstream sorting of the population of interest. Our group has reported that the addition of synthetic peptide pools of all predicted class-I neoantigens can improve conventional TIL generation in ovarian cancer (20). Primed TIL cultures were significantly enriched in neoepitope-specific CD8^+^ T-cells as compared with standard TILs generated from the same patients.

Finally, while it is tempting to focus on private neoantigens deriving from single point mutations, driver genes (such as RAS and BRAF) recurrently affected by mutation or fusion events across individuals and cancer types would be expected to yield semiprivate (or even “shared”) neoantigens. This seems to be especially the case for hematological malignancies, where immunogenic neoantigens have been reported to be mutated in up to 30% of patients (125, 126), along with case reports of mutation hot spots in solid tumors (127–130). In addition, potential semiprivate neoantigens derived from aberrant phosphorylation, resulting from dysregulated protein kinase activity during transformation, can be detected with mass spectrometry using relatively small amounts of patient samples (9, 131, 132). The targeting of shared or semiprivate neoantigens in solid tumors is a particularly desirable possibility which has to be further investigated, considering it would contribute to greatly reducing costs and production time of highly enriched T-cell infusion products. Given the multistep and laborious nature of these enriched T-cell therapies, which require coordination between highly specialized healthcare centers and manufacturing cell facilities, one has to ultimately consider if the added time frame is clinically reasonable. An additional month to the pipeline can result in significant patient dropout because of rapid disease progression (133).

## Concluding Remarks

The identification of neoantigens as drivers of successful antitumor immunity is offering exciting new opportunities for cancer immunotherapies, including making T-cell infusion products highly individualized for more effective treatment. Moving forward, patient-specific T-cell enrichment technologies will need to be integrated into clinically compliant pipelines. In this respect, the first Food and Drug Administration (FDA) approval of the first adoptive cell therapy [i.e., chimeric antigen receptor (CAR) T-cell therapy] represents a huge achievement in the immune-oncology field and will hopefully pave the way for further approvals of personalized immunotherapies.

## Author Contributions

VB wrote the manuscript and made the figures. VB, AH, and GC contributed to the concept, discussion of content, and editing of the manuscript. All authors contributed to the article and approved the submitted version.

## Conflict of Interest

The authors declare that the research was conducted in the absence of any commercial or financial relationships that could be construed as a potential conflict of interest.
